# Evolution of adolescents’ dietary patterns in Northeast Brazil from 2008 to 2018

**DOI:** 10.11606/s1518-8787.2024058005090

**Published:** 2024-02-09

**Authors:** Soraia Pinheiro Machado, Ilana Nogueira Bezerra, Mariane Alves Silva, Maria Helena Lima D’oran, Diana Barbosa Cunha, Luis Alberto Moreno, Rosely Sichieri

**Affiliations:** I Universidade Estadual do Ceará Centro de Ciências da Saúde Fortaleza CE Brazil Universidade Estadual do Ceará. Centro de Ciências da Saúde. Fortaleza, CE, Brazil; II Universidade Federal de Mato Grosso Faculdade de Nutrição Cuiabá MT Brazil Universidade Federal de Mato Grosso. Faculdade de Nutrição. Cuiabá, MT, Brazil; III Universidade do Estado do Rio de Janeiro Departamento de Epidemiologia Rio de Janeiro RJ Brazil Universidade do Estado do Rio de Janeiro. Departamento de Epidemiologia. Rio de Janeiro, RJ, Brazil; IV Universidad de Zaragoza Facultad de Ciencias de la Salud Zaragoza Spain Universidad de Zaragoza. Facultad de Ciencias de la Salud. Zaragoza, Spain

**Keywords:** Adolescent, Eating

## Abstract

**OBJECTIVE:**

To evaluate the evolution of the dietary patterns of adolescents in the northeast region of Brazil.

**METHODS:**

Secondary analysis of data from the *Pesquisa de Orçamentos Familiares* (POF – Household Budget Surveys), collected by the Brazilian Institute of Geography and Statistics (IBGE) in the years 2008–2009 and 2017–2018. A total of 3,095 adolescents were evaluated in 2008–2009 and 3,015 in 2017–2018. Food consumption was assessed using two dietary records in 2008–2009 and two 24-hour recalls in 2017–2018, applied on non-consecutive days. Based on these data, principal components factor analysis (PCFA) was performed, followed by orthogonal rotation of the varimax type, to derive dietary patterns, stratified by sex. The results were described as means or percentage frequencies, with their respective 95% confidence intervals.

**RESULTS:**

Three main dietary patterns were identified among adolescents from the northeast region of Brazil. Among boys, in 2008–2009, the patterns were called *snacks, traditional Brazilian*, and *coffee*; and in 2017–2018, *traditional Brazilian, snacks*, and *mixed*, in this order of representativeness of the group’s eating habits. Among female adolescents, in 2008–2009, the patterns were *snacks, traditional Brazilian*, and *coffee*; and in 2017–2018, *traditional Brazilian, snacks*, and *processed meats*.

**CONCLUSION:**

The dietary patterns identified in 2008–2009 and 2017–2018 were similar in both genders; however, the *snacks* pattern, which explained most of the data variability in 2008–2009, was replaced by the *traditional Brazilian*.

## INTRODUCTION

The food consumption pattern of the Brazilian population has changed in recent years, with higher consumption of ultra-processed foods and a decrease in the consumption of fresh foods^1^. These changes are associated with an increase in chronic non-communicable diseases (NCDs) in all age groups, including adolescence^2^.

Adolescence is characterized by a phase marked by intense body changes, with increased energy and nutritional needs^3^, an unhealthy lifestyle, and greater susceptibility to environmental influences^4^. In this age group, breakfast is often skipped and dinner is replaced by snacks, with high consumption of foods characteristic of an unhealthy diet^5^. On the other hand, a study that evaluated the temporal variation of the diet in this population from 2009 to 2015, based on data from the *Pesquisa Nacional de Saúde do Escolar* (PeNSE – National Adolescent School-based Health Survey), identified a decrease in the regular consumption of beans, candy/sweets, and soft drinks and an increased consumption of vegetables^6^. Regarding adolescents from the northeast region, due to its distinctive cuisine, a study identified the presence of dietary patterns with typical regional dishes based on corn and cassava^7^.

Among adolescents in the northeast region, this scenario is even more concerning, since this is the region with the highest prevalence of food insecurity in Brazil, also presenting the worst conditions of employment, income, and schooling among the families^8^. Food insecurity was inversely associated with adherence to the “*Mediterranean*” dietary pattern (consisting of fruit, vegetables, and whole foods) among adolescents^9^. However, studies on this topic are scarce^5,6^. Assessing the dietary profile of adolescents in this region can contribute to the development of obesity prevention programs^10^.

The *Pesquisa de Orçamentos Familiares* (POF – Household Budget Survey) is a sample survey whose objective is to measure the food consumption of individuals. To this end, the *Inquéritos Nacionais de Alimentação* (INA – National Food Survey) is carried out with a subsample of the POF. The last edition of the survey took place in 2017–2018 and, therefore, the POF is the most complete and up-to-date database in the country^11^. It should be noted that the sampling process of the 2017–2018 POF maintained the same characteristics of the design applied to the 2008–2009 POF, allowing the comparability of data and the analysis of the dietary evolution of individuals^11,12^.

Therefore, this study aims to evaluate the evolution of the food consumption pattern of adolescents in the northeast region of Brazil, from 2008 to 2018.

## METHODS

This study is a secondary analysis of data from the INA^11,12^, carried out on subsamples from the last two editions of the 2008–2009 and 2017–2018 POFs, by the *Instituto Brasileiro de Geografia e Estatística* (IBGE – Brazilian Institute of Geography and Statistics). As this is a study with secondary data, approval by the ethics committee was waived.

Both surveys were carried out on representative samples of the Brazilian population, generating estimates for the five major regions, urban and rural areas, and different socioeconomic levels. For sample selection, a common sample, or master sample, was used in both POFs, designed by the IBGE based on the *Sistema Integrado de Pesquisas Domiciliares* (SIPD – Integrated System of Household Surveys). A cluster sampling plan was used, divided into two stages. The primary sampling units (PSUs) were derived from the master sample, which consists of a set of census sectors. The PSUs were selected by sampling with probability proportional to the number of households in each sector. In the second stage, the households were selected by simple random sampling. The sectors were distributed over the four quarters of the year in which the research was carried out. Details of the methodology can be found in IBGE (2011)^11^, and IBGE (2019)^12^.

In the 2008–2009 POF, 55,970 households were selected. As for the INA, 16,764 households were selected, in which individuals aged 10 years or older answered the food consumption survey. The total number of respondents was 34,003, of which 12,615 lived in the urban area of northeastern Brazil. This study evaluated adolescents aged 10 to 19 years (3,032) living in the urban area of the northeast region, excluding pregnant and lactating women (81). Therefore, the final sample consisted of 2,951 adolescents in the 2008–2009 INA.

In the 2017–2018 POF, the total number of selected households was 57,920. For the INA, 20,112 households with 46,164 individuals aged 10 years and older participated in the survey. Of these, 16,097 lived in the urban area of the northeast region. In this study, adolescents from the northeast region aged from 10 to 19 years (3,095) were evaluated, excluding pregnant and lactating women (80), resulting in a final sample of 3,015 adolescents in the 2017–2018 INA.

For this study, all adolescents from the Northeast region sampled in the two editions of the INA were considered (2,951 from the 2008–2009 database and 3,015 from the 2017–2018 group).

In the 2008–2009 INA, participants filled out the food records for two non-consecutive days, recording all types and quantities of food and beverages (except water), their preparation methods for some foods, and the time and place where the food was consumed. The respondents were given a booklet with instructions on how to fill out the records, which contained photos of household measuring tools and portion sizes to facilitate estimating the amount of food consumed. All records were reviewed by the interviewers to assess missing information and correct recording errors.

In the 2017–2018 INA, two 24-hour recalls were applied on non-consecutive days, following the multiple-step method, with detailed information on the amount of food consumed, time and places of consumption, and occasion of meals, with data on how the food was prepared only required for specific foods. In this study, data from the first day of consumption were used. In the 2017–2018 survey, data on “additional items” (olive oil, butter/margarine, sugar, sweeteners, honey, molasses, mayonnaise, ketchup, mustard, soy sauce, shredded cheese, and heavy cream), which are commonly consumed together with bread, pasta, among others, were obtained when the individual reported consuming any of these foods.

For this study, the first day of food consumption was considered in both surveys. To estimate the amount consumed by the individuals, the measurement tables used for foods consumed in Brazil were used, obtained from the 2008–2009 INA and the 2017–2018 INA, with their respective amounts reported in grams or milliliters.

Daily consumption, in grams/milliliters, of the aforementioned food items was used as a dietary variable for the analysis to identify dietary patterns in this study. For this purpose, the food items mentioned (1,121 items in 2008–2009 and 1,593 in 2017–2018) were grouped by the researchers according to the nutritional composition in each period, totaling 14 food groups for the two surveys: rice (white rice, brown rice, and rice-based preparations), bread and pasta (white and whole grain bread, toast, croissant, pasta items, pasta-based preparations, and instant noodles), beans (beans, green beans, chickpeas, lentils, peas, and meat substitutes), fruit and vegetables (all fruit except fruit and vegetable/green vegetable juices in general), roots and derivatives (sweet potatoes, cassava, yams, mushrooms, soufflés, potatoes and potato-based preparations, preparations with cassava flour, tapioca starch, corn, and corn-based preparations), soups (broths, soups, chicken soup, and bisque), cakes and biscuits (cakes, cookies, biscuits, cereal bars, baked and fried snacks, savory pastries, potato/corn chips, and crackers), processed meats (ham, bacon, bologna, salami, cold cuts, and frankfurters), milk and dairy products (milk, smoothies, yogurt, curds, fermented milk, mozzarella, cottage, cheddar, cream cheese, and others—except dairy-based desserts), sweets/candy (pies, candy, milk and fruit-based desserts, sweet pastries, chocolate—including sweets and chocolate beverages), coffee (coffee and tea), snacks (pizzas, hamburgers, cheeseburgers, hot dogs and others, potato/corn chips, crackers, baked and fried snacks, savory pastries), meat, chicken, fish, and eggs (beef, pork, beef/pork dishes, eggs and preparations with eggs, fish, shrimp, crab and preparations with fish/shellfish, and chicken and chicken-based preparations) and juices and soft drinks (fruit juices, *cajuína* [juice of cashew apples], coconut water, soft drinks, artificial juices, sugarcane juice, regular and diet/light soft drinks, energy drinks). For this study, the additional items from the 2017–2018 POF were only included when they were mentioned spontaneously by the respondents, so that the collection methodology was as similar as possible to that of 2008–2009.

The dietary patterns were obtained, stratified by gender, using the Principal Component Factor Analysis (PCFA) followed by varimax orthogonal rotation. The Kaiser-Meyer-Olkin coefficient (KMO ≥ 0.5) and Bartlett’s sphericity test (p < 0.05) were used to test the applicability of factor analysis to food consumption data. The Cattel plot (scree plot) was used as a criterion to define the number of factors retained and the conceptual meaning of the patterns identified in the analyses that retained beyond the number indicated by the Cattel plot (nCattel), nCattel - 1, and nCattel + 1. No components with eigenvalues > 1.0 were retained and food groups that showed a factor loading value ≥ 0,30 were included in the dietary pattern. Food items with positive loads are close to the pattern, that is, they are foods consumed more frequently in that pattern, whereas items with negative loads are far from the pattern, indicating that the food is not often consumed in that pattern. It was decided to also keep the food items with negative factor loadings in the patterns. The food patterns identified were named according to the composition of their food items, choosing, whenever possible, nomenclatures already established in the literature.

The results were described as means for continuous variables or percentage frequencies for categorical variables, with their respective 95% confidence intervals, considering the complexity of the study design and the sampling weights. Statistical analyses were performed using Stata software, version 15.0.

## RESULTS

The adolescents’ mean age was 14.5 years in both periods, they were mostly males (53.1% in 2008–2009 and 53.3% in 2017–2018), of black and mixed ethnicity (74.7% in 2008–2009 and 75.3% in 2017–2018). Regarding the level of schooling of the head of the family, the highest prevalence comprised individuals who had studied up to four years (55.3% in 2008–2009 and 36.1% in 2017–2018). There was an increase in the prevalence of adolescents enrolled in public schools (78.3% in 2008–2009 and 87.4% in 2017–2018) and private schools (8.5% in 2008–2009 and 12.6% in 2017–2018), and a decrease in the mean number of residents in the household (5.1 and 4.7) (Table 1).

**Table 1:** Characterization of adolescents from the Northeast region evaluated in the 2008–2009 and 2017–2018 INAs.

Characteristic	2008–2009 INA	2017–2018 INA
	
	(n = 2,951)	(n = 3,015)
	
	m (95%CI)	m (95%CI)
Age (mean)	14.5 (14.3–14.6)	14.5 (14.4–14.7)
Gender		
Male	53.1 (50.6–55.7)	53.3 (51.2–55.4)
Female	46.9 (44.3–49.4)	46.7 (44.6–48.8)
Ethnicity		
White	24.5 (22.1–26.8)	24.1 (21.8–26.3)
Black and mixed	74.7 (72.4–77.1)	75.3 (73.1–77.6)
Asians and Indigenous	0.8 (0.2–1.3)	0.6 (0.2–1.0)
Head of the family’s level of schooling (years)		
> 4		
4–8	55.3 (52.2–58.5)	36.1 (33.2–39.0)
9–11	20.5 (17.8–23.3)	25.1 (22.5–27.8)
≥ 12	14.9 (12.7–17.1)	12.4 (10.5–14.4)
	9.2 (7.5–11.0)	26.3 (23.9–28.8)
Type of school attended		
Public	78.3 (76.0–80.7)	87.4 (85.7–89.1)
Private	8.5 (6.9–10.0)	12.6 (10.9–14.3)
Number of household residents (mean)	5.1 (4.9–5.2)	4.7 (4.5–4.8)

INA: *Inquéritos Nacionais de Alimentação* (National Food Survey); 95%CI: 95% confidence interval; m: mean.

When comparing the frequencies and averages of consumption (grams/days) of the food groups in 2008–2009 and 2017–2018, a higher consumption of snacks was observed. On the other hand, there was a lower consumption of fruit and vegetables, milk and dairy products, sweets, and coffee (Table 2).

**Table 2:** Frequencies (%), consumption averages (grams/day) and 95% confidence intervals of foods and food groups in adolescents living in the northeast region of Brazil, according to data from the 2008–2009 and 2017–2018 POFs.

Food groups	2008–2009 INA		2017–2018 INA	
	
	Frequency (95%CI)	m (95%CI)	Frequency (95%CI)	m (95%CI)
1. Rice	85.6 (83.5–87.7)	155.1 (145.7–164.5)	85.1(83.2–86.9)	144.8 (137.4–152.2)
2. Bread and pasta	65.5 (62.5–68.4)	102.6 (95.2–110.0)	61.9 (59.2–64.5)	107.3 (100.0–114.6)
3. Beans	78.0 (75.3–80.7)	188.5 (175.7–201.4)	78.3 (76.2–80.5)	178.4 (167.5–189.3)
4. Fruit and vegetables	36.8 (33.9–39.6)	82.5 (72.2–92.9)	29.1 (26.8–31.3)	51.1 (46.2–56.0)
5. Roots and derivatives	50.3 (47.3–53.3)	82.0 (74.0–90.0)	53.0 (50.0–55.9)	87.8 (79.6–95.9)
6. Soups	12.1 (10.3–13.9)	51.4 (43.0–59.9)	9.1 (7.5–10.6)	41.0 (33.6–48.4)
7. Cakes and biscuits/cookies	47.4 (44.4–50.4)	41.9 (37.7–46.1)	52.8 (50.2–55.5)	46.4 (43.2–49.7)
8. Meat, chicken, fish, and eggs	85.1 (82.9–87.3)	147.0 (137.2–156.7)	86.8 (84.8–88.8)	149.5 (142.1–157.0)
9. Processed meats	17.1 (14.8–19.4)	15.3 (11.7–18.9)	19.2 (17.2–21.3)	15.4 (12.9–17.8)
10. Milk and dairy products	28.5 (25.9–31.1)	77.9 (66.2–89.7)	18.0 (16.0–19.9)	44.9 (37.9–48.2)
11. Sweets/candy	32.7 (30.1–35.2)	50.8 (43.9–57.7)	23.3 (21.1–25.6)	42.8 (37.4–48.2)
12. Juices and soft drinks	55.8 (52.7–58.8)	226.2 (211.1–241.4)	58.8 (55.9–61.8)	245.8 (230.5–261.0)
13. Coffee	76.9 (74.6–79.2)	203.6 (191.8–215.3)	69.5 (67.1–71.9)	131.5 (119.0–144.1)
14. Snacks	19.9 (17.8–22.0)	22.0 (18.8–25.1)	25.3 (22.8–27.8)	40.5 (34.8–46.2)

POF: *Pesquisa de Orçamentos Familiares* (Household Budget Survey); INA: *Inquéritos Nacionais de Alimentação* (National Food Survey); 95%CI: 95% confidence interval; m: mean.

Note: values in bold indicate statistical significance.

For the two periods analyzed, three main dietary patterns were retained among male and female adolescents. In 2008–2009, the main dietary patterns identified in this study were *snacks* (bread and pasta, cakes and cookies, sweets, juices and soft drinks, and fast-food snacks); *traditional Brazilian* (rice, beans, meat, chicken, fish, and eggs, with negative factor loading for bread and pasta); and *coffee* (roots and derivatives and coffee, with negative factor loadings for soups, milk, and dairy products). In 2017–2018, the main dietary patterns identified were *traditional Brazilian* (rice, beans, meat, chicken, fish, eggs, and coffee); *snacks* (cakes and biscuits/cookies, juices and soft drinks, and fast-food snacks); and *mixed* (roots and derivatives, soups, and processed meats; and negative factor loading for bread and pasta) (Table 3).

**Table 3:** Distribution of factor loadings of foods in the main food consumption patterns of male adolescents living in the northeast region of Brazil, according to POF data from the years 2008–2009 and 2017–2018.

Foods and food groups	Dietary patterns			Dietary patterns		
	
	2008–2009 INA			2017–2018 INA		
	
	Snacks	Traditional Brazilian	Coffee	Traditional Brazilian	Snacks	Mixed
1. Rice		0.75		0.76		
2. Bread and pasta	0.42	-0.32				-0.30
3. Beans		0.58		0.55		
4. Fruit and vegetables						
5. Roots and derivatives			0.68			0.72
6. Soups			-0.34			0.48
7. Cake and biscuits/cookies	0.39				0.57	
8. Meat, chicken, fish, and eggs		0.45		0.47		
9. Processed meats						0.58
10. Milk and dairy products			-0.31			
11. Sweets/candy	0.34					
12. Juices and soft drinks	0.77				0.75	
13. Coffee			0.64	0.57		
14. Snacks	0.65				0.56	
% of variability explanation	13.17	9.82	8.79	11.97	9.63	8.67

POF: *Pesquisa de Orçamentos Familiares* (Household Budget Surveys); INA: *Inquéritos Nacionais de Alimentação* (National Food Survey).

Note: dietary patterns are presented in descending order of representativeness, expressed by the percentage of variance explained in each pattern.

Among female adolescents, in 2008–2009, the patterns were called *snacks* (cakes and biscuits/cookies, sweets/candy, juices and soft drinks, and fast-food snacks); *traditional Brazilian* (rice, beans, meat, chicken, fish, and eggs, with negative factor loading for bread and pasta); and *coffee* (roots and derivatives, processed meats, and coffee, with negative factor loading for soups, milk, and dairy products). In 2017–2018, the main dietary patterns were named *traditional Brazilian* (rice, beans, meat, chicken, fish, eggs, and coffee, with a negative factor loading for bread and pasta); *snacks* (cakes and biscuits/cookies, sweets/candy, juices and soft drinks, and fast-food snacks, with a negative factor loading for coffee); and *processed meats* (processed meats and coffee, with negative factor loadings for meat, chicken, fish, eggs, and fruits and vegetables) (Table 4).

**Table 4:** Distribution of factor loadings of foods in the main food consumption patterns among female adolescents living in the northeast region of Brazil, according to POF data from 2008–2009 and 2017–2018.

Characteristics	Dietary patterns			Dietary patterns		
	
	2008–2009 INA			2017–2018 INA		
		
Foods and food groups	Snacks	Traditional Brazilian	Coffee	Traditional Brazilian	Snacks	Processed meats
1. Rice		0.64		0.72		
2. Bread and pasta		-0.44		-0.32		
3. Beans		0.33		0.74		
4. Fruits and vegetables						-0.30
5. Roots and derivatives			0.60			
6. Soups			-0.34			
7. Cake and biscuits/cookies	0.44				0.38	
8. Meat, chicken, fish, and eggs		0.68		0.30		-0.61
9. Processed meats			0.36			0.67
10. Milk and dairy products			-0.44			
11. Sweets/candy	0.43				0.45	
12. Juices and soft drinks	0.73				0.64	
13. Coffee			0.53		-0.40	0.33
14. Snacks	0.60				0.55	
% of variability explanation	12.17	9.43	8.58	11.51	9.53	8.69

POF: *Pesquisa de Orçamentos Familiares* (Household Budget Survey); INA: *Inquéritos Nacionais de Alimentação* (National Food Survey).

Note: dietary patterns are presented in descending order of representativeness, expressed by the percentage of variance explained in each pattern.

From 2008 to 2018, the pattern with the greatest representation in the feeding behavior of adolescents was replaced. In 2008–2009, the *snack* pattern was the one that contributed most to the proportional variance, and in 2017–2018, in both genders, it was replaced by the *traditional Brazilian* pattern.

## DISCUSSION

Based on the two 2008–2009 and 2017–2018 POFs, we identified three main dietary patterns among adolescents in the northeast region of Brazil. Among boys, in 2008–2009, the patterns were called *snacks, traditional Brazilian*, and *coffee*; and in 2017–2018, *traditional Brazilian, snacks*, and *mixed*, following this order of representativeness of the group’s eating habits. Among female adolescents, in 2008–2009, the patterns were called *snacks, traditional Brazilian*, and *coffee*; and in 2017–2018, *traditional Brazilian, snacks*, and *processed meats*.

The food consumption pattern called *traditional Brazilian* was found in both periods evaluated and in both genders. These results corroborate other national studies^13,14^, including those carried out with a sample of adolescents from the northeast region^15^.

In 2017–2018, the *traditional Brazilian* pattern appeared as the most representative among adolescents, replacing the *snacks* food pattern, which appeared in this position in 2008–2009. A study has already shown an inverse relationship between this dietary pattern and excess weight^16^, highlighting the nutritional benefits of consuming rice and beans as a source of iron, folate, fibers, and good quality proteins^17^. First, seen as a beneficial change in terms of feeding behavior, the replacement of the pattern *snacks* by the *traditional Brazilian* may be a reflection of the economic and social crisis that Brazil has been facing since 2014, with the northeastern states being the most affected. This condition contributes to an increase in food consumption at a lower cost^18^. A study carried out with 1,139 Brazilian adolescents revealed that individuals of lower socioeconomic status were more likely to adhere to the “*traditional*” pattern, as it consists of foods that are easily acquired and less expensive^13^. A reflection of this reality is the decrease in the factor loading of the meat group in the second survey, from 0.68 in 2008–2009 to 0.30 in 2017–2018. It should also be noted that there was an increase in the level of schooling of the head of the family. The level of schooling and, consequently, the parents’ income are factors that directly influence the dietary pattern of adolescents^19^, which may have contributed to this change in the individuals’ dietary patterns.

Regardless of the causal factor behind the decrease in meat consumption, in biological terms, this reduction can positively affect the adolescents’ health in the short and long term and sustainability. Excessive meat consumption is related to colorectal cancer and cardiovascular diseases^20^. It should be noted that livestock farming impacts heavily on the environment and is considered one of the main contributors to global warming. In addition to greenhouse gas emissions, meat production is associated with pollution and water scarcity. Corroborating our results, a review study identified that young individuals are among the consumers with the greatest environmental concerns and, consequently, those who most limit their meat consumption^21^. However, it should be remembered that the excessive consumption of red meat has been evidenced as a risk factor, and the daily consumption of meat by Brazilian adolescents in 2017–2018 was 46.4 g^11^, that is, far from excessive consumption. Moreover, the changes in food consumption between the two surveys impacted negatively on the prevalence of inadequate iron intake^11,12^. In addition, the “meat” group of this study combines foods such as eggs, meat, and fish that accompany the combination of rice and beans, and is the main source of protein in the diet. Therefore, overall, reducing its consumption should be viewed with caution.

The *snacks* pattern, also present in both genders and in the two periods evaluated, was characterized by the presence of bread, pasta, cakes, biscuits/cookies, juices, soft drinks, and fast-food snacks. This pattern was also found in other Brazilian study that evaluated the same age group^14^. Among Brazilian adults participating in the same surveys, a similar pattern was also found by Antunes et al.^22^ and was called the “*Western*” pattern. The consumption of these food groups has increased in recent decades, since consuming ultra-processed foods is easy and practical for consumers^23^. However, the Dietary Guideline for the Brazilian Population discourages the consumption of these foods^24^, as they are markers of an unhealthy diet^25^ and are associated with the onset of several diseases, such as obesity, hypertension, and dyslipidemia^2,26,27^.

In the last PeNSE, carried out in 2019, it was observed that the diet of adolescents is characterized by the maintenance of consumption of traditional foods, such as beans, but with a high consumption of ultra-processed foods^28^, similar to the *mixed* pattern observed in 2017–2018. This pattern replaced the *coffee* pattern, which previously consisted of roots and derivatives, coffee, meat, chicken, fish, and eggs and held a negative factor loading for soups, milk, and dairy products. In 2017–2018, the *mixed* pattern showed a change in the positive factor loading for soups, the inclusion of processed meats, and the elimination of the coffee, meat, chicken, fish, and eggs group. The substitution of meat, chicken, fish, and eggs for processed meats reinforces the reality of current food consumption, with an increase in the consumption of ultra-processed foods to the detriment of a decrease in the consumption of fresh and minimally processed foods^1^. In addition, as already mentioned, the northeastern states are among those most affected by the socioeconomic crisis^18^ and protein foods such as meat, chicken, and fish are the most costly in the budget. In both the 2008–2009 and 2017–2018 POFs, the meat, viscera, and fish group contributed the most to the family’s average monthly monetary and non-monetary expenditure^11,12^. In the northeast region, 22.3% of families’ expenditure on food came from this group^14^.

It was observed that, among boys, the *coffee* pattern was identified in the first survey, consisting of coffee and roots and derivatives, whereas, in 2017–2018, the food item coffee made up the *traditional Brazilian* pattern, the pattern with the highest representation in the period evaluated. Adolescents are the age group with the greatest exposure to the media and social networks and, especially among males, there is a greater search for hypertrophy. A progressive increase in caffeine intake among adolescents has already been observed, with the aim of improving performance in physical exercise^29^.

Among girls, the *coffee* pattern also underwent changes between the two periods evaluated. In the 2008–2009 period, the pattern consisted of roots and derivatives, processed meats, and coffee, with a negative factor loading for soups, milk, and dairy products. In the 2017–2018 period, the pattern consisted of processed meats and coffee, with a negative factor loading for meat, chicken, fish, eggs, fruits, and vegetables and was therefore called *processed meat* pattern. The replacement of healthy foods, such as roots and derivatives, by foods with high energy density and low nutritional value, such as processed meats, is a common behavior in adolescence^30^.

When comparing the frequencies and means of consumption of food groups in the years 2008–2009 and 2017–2018, there was a reduction in the consumption of fruits and vegetables, milk and derivatives, sweets, and coffee. The lower consumption of sweets can be attributed to the Dietary Guideline recommendation for the Brazilian population, which encourages the consumption of fresh and minimally processed foods and discourages the consumption of processed and ultra-processed foods^24^. On the other hand, adolescents are known to be a risk group for skipping breakfast^5^ and replacing healthy foods^30^. Such attitudes may contribute to reducing the consumption of milk and derivatives, coffee, fruits, and vegetables.

Adolescence is a period marked by learning, so it is essential to encourage the development and teaching of culinary skills, as recommended by the Dietary Guidelines for the Brazilian Population, and to share household activities related to food preparation^24^. Lack of cooking skills was a determining factor in the consumption of ready meals and ultra-processed foods^31^.

Eating habits acquired in childhood and adolescence are known to be perpetuated in adulthood^32^; therefore, the evolution of the dietary pattern of adolescents from the northeast region confirms the need for recommendations aimed at increasing the consumption of fresh and minimally processed foods and reducing the consumption of ultra-processed foods. Especially in this region, which is one of the most affected by the current economic crisis, it is necessary to adopt public policies that enable the population to access these healthy foods. Currently, fresh and minimally processed foods are significantly less expensive than ultra-processed foods^33^; however, there are projections showing that, from 2026 onwards, healthy eating will become more expensive than the unhealthy type^34^. Thus, it is important to adopt measures that can prevent this inversion of values.

The limitation of this study was the difference in the dietary surveys used to assess food consumption. The 2008–2009 INA evaluated consumption using the food record, whereas the 2017–2018 INA used the 24-hour recall to evaluate food consumption. However, in both periods, the multiple-step technique was applied, reviewing the record and during recalls, which may have minimized possible methodological differences. It is noteworthy that there have been studies evaluating the dietary pattern of adolescents in the northeast region of Brazil; however, the samples were small. This is a population-based study, with a representative sample of the region. Moreover, the 2017–2018 POF is considered the most complete database research of this type in the country and is also the most up-to-date^14^.

In conclusion, this study identified three main dietary patterns among adolescents in the northeast region of Brazil. Among boys, in 2008–2009, the patterns *snacks, traditional Brazilian*, and *coffee* were observed; and in 2017–2019, the *traditional Brazilian, snacks*, and *mixed* patterns were observed, following this order of representativeness of the group’s feeding behavior. Among females, in 2008–2009, the patterns were called *snacks, traditional Brazilian*, and *coffee*; and in 2017–2018, *traditional Brazilian, snacks*, and *processed meats*. Although the dietary patterns identified in 2008–2009 and 2017–2018 were similar for both sexes, the *snack* pattern, which best explained the data variability in 2008–2009, was replaced by the *traditional Brazilian*.
